# Morbidity management and disability prevention for lymphatic filariasis in Sri Lanka: Current status and future prospects

**DOI:** 10.1371/journal.pntd.0006472

**Published:** 2018-05-10

**Authors:** Nilmini Chandrasena, Ranjan Premaratna, Indeewarie. E. Gunaratna, Nilanthi R. de Silva

**Affiliations:** 1 Department of Parasitology, Faculty of Medicine, University of Kelaniya, Kelaniya, Sri Lanka; 2 Department of Medicine, Faculty of Medicine, University of Kelaniya, Kelaniya, Sri Lanka; 3 Anti Filariasis Campaign, Ministry of Health, Colombo, Sri Lanka; RTI International, UNITED STATES

## Abstract

**Background:**

Sri Lanka was acknowledged to have eliminated lymphatic filariasis (LF) as a public health problem in 2016, largely due to its success in Mass Drug Administration (MDA) to interrupt disease transmission. Analysis of the Strengths, Weaknesses, Opportunities and Threats (SWOT) of the national Morbidity Management and Disability Prevention (MMDP) program, the other pillar of the LF control program, was carried out with the objective of evaluating it and providing recommendations to optimize the use of available resources.

**Methodology:**

A situation analysis of the MMDP activities provided by the state health sector was carried out using published records, in-depth interviews with key informants of the Anti Filariasis Campaign, site-visits to filariasis clinics with informal discussions with clinic workforce and personal communications to identify strengths and weaknesses; and opportunities to overcome weaknesses and perceived threats to the program were explored.

The principal strength of the MMDP program was the filariasis clinics operational in most endemic districts of Sri Lanka, providing free health care and health education to clinic attendees. The weaknesses identified were the low accessibility of clinics, incomplete coverage of the endemic region and lack of facilities for rehabilitation. The perceived threats were diversion of staff and resources for control of other vector-borne infections, under-utilization of clinics and non-compliance with recommended treatment. Enhanced high level commitment for MMDP, wider publicity and referral systems, integration of MMDP with other disease management services and collaboration with welfare organizations and research groups were identified as opportunities to overcome weaknesses and challenges.

**Conclusions:**

The recommended basic package of MMDP was functional in most of the LF-endemic region. The highlighted weaknesses and challenges, unless addressed, may threaten program sustainability. The identified opportunities for improvement of the programme could ensure better attainment of the goal of the MMDP program, namely access to basic care for all affected by lymphatic filarial disease.

## Author summary

Lymphatic filariasis (LF) is a tropical disease causing swelling of limbs (lymphoedema, elephantiasis) and male genitalia (hydrocele). It is a disabling and deforming disease caused by parasitic worms transmitted by mosquitoes. The Sri Lankan Anti Filariasis Campaign was successful in reducing LF transmission to very low levels by annual mass drug administration, resulting in recent achievement of elimination status. The other pillar of LF elimination is the management of morbidity and disability prevention (MMDP) among those with lymphatic filarial disease. The strengths, weaknesses and threats of the MMDP program were evaluated and opportunities for improvement were explored in June 2017. The filariasis clinics established in the endemic area, providing care for patients with lymphoedema and elephantiasis, were identified as the main strength. Hospital surgical units were important in the treatment of hydrocele. The weaknesses were low accessibility of clinics, incomplete coverage of endemic districts, lack of facilities for rehabilitation and lack of morbidity targets. The threats were diversion of staff and resources, under-utilization of clinics, social stigma and loss of alliances. Enhanced commitment to improve coverage and access to clinics in the endemic areas, wider publicity, referral systems, integration of MMDP into other disease management services, collaborations with welfare organizations and research groups were the opportunities identified.

## Introduction

LF is an important public health and socioeconomic problem worldwide. It has been ranked as the second leading cause of permanent disability [1]. Of the disease manifestations associated with LF, the most distressing and disabling are lymphoedema, its advanced form elephantiasis, hydrocele and acute inflammatory episodes termed dermatolymphangioadenitis (ADLA) caused by secondary infection of lymphoedematous tissues. Physical and social functions as well as psychological wellbeing of patients with lymphoedema and elephantiasis are significantly impaired due to the pain and discomfort, restricted mobility, social stigmatization, feelings of embarrassment and emotional distress that accompany these chronic disfiguring manifestations [2, 3, 4]. Those afflicted with advanced disease may lose their livelihoods due to the associated disability or social stigma [5]. The ADLA episodes are extremely painful and incapacitating, incurring a significant financial burden in the form of direct and indirect costs attributed to medication and lost income [6, 7, 8, 9, 10]. It is estimated that globally, 120–129 million people are infected with LF and of these, around 40 million have overt disease, accounting for 5.9 million disability adjusted life years (DALYs), with a concomitant loss of productivity and social stigmatization [11]. Therefore, LF was identified as a major public health problem and is targeted by the World Health Organization (WHO) for elimination by 2020.

The Global Program for Eliminating LF (GPELF) launched in year 2000, consists of two main components: MDA to stop the spread of infection and MMDP to manage chronic disease. MDA for LF involves the annual provision of a combined dose of medications {diethylcarbamazine citrate (DEC) + albendazole (old regimen) + ivermectin (new triple therapy) or in areas co-endemic for onchocerciasis, ivermectin + albendazole} to all eligible persons living in an endemic area, for at least 5 years. MMDP involves a basic package of recommended health-care services to alleviate suffering and prevent further progression of disease [12]. The minimum recommended package for MMDP includes i) MDA, which in addition to reducing microfilariaemia below a target threshold at which transmission is considered non-sustainable, may also destroy invading larvae (L3) or juvenile worms in those with recently acquired infections, or selective treatment of individuals positive for infection with a prolonged regimen of DEC to destroy any remaining adult parasites or microfilaria; ii) surgery for hydrocele; iii) treatment of ADLA; iv) prevent progression of lymphoedema and ADLA [13].

Considerable progress has been made with regard to MDA. Of 73 countries considered to be endemic in 2014, 20 had progressed to the post-MDA surveillance phase by 2016 and 52 required further rounds of MDA [14]. However, unlike MDA, only 34 endemic countries have initiated MMDP services and there is evidence that coverage is being monitored by implementation units in only 25 countries [14]. Therefore, implementation of MMDP appears to be far behind MDA in most countries [15].

### LF in Sri Lanka

**Fig 1:**
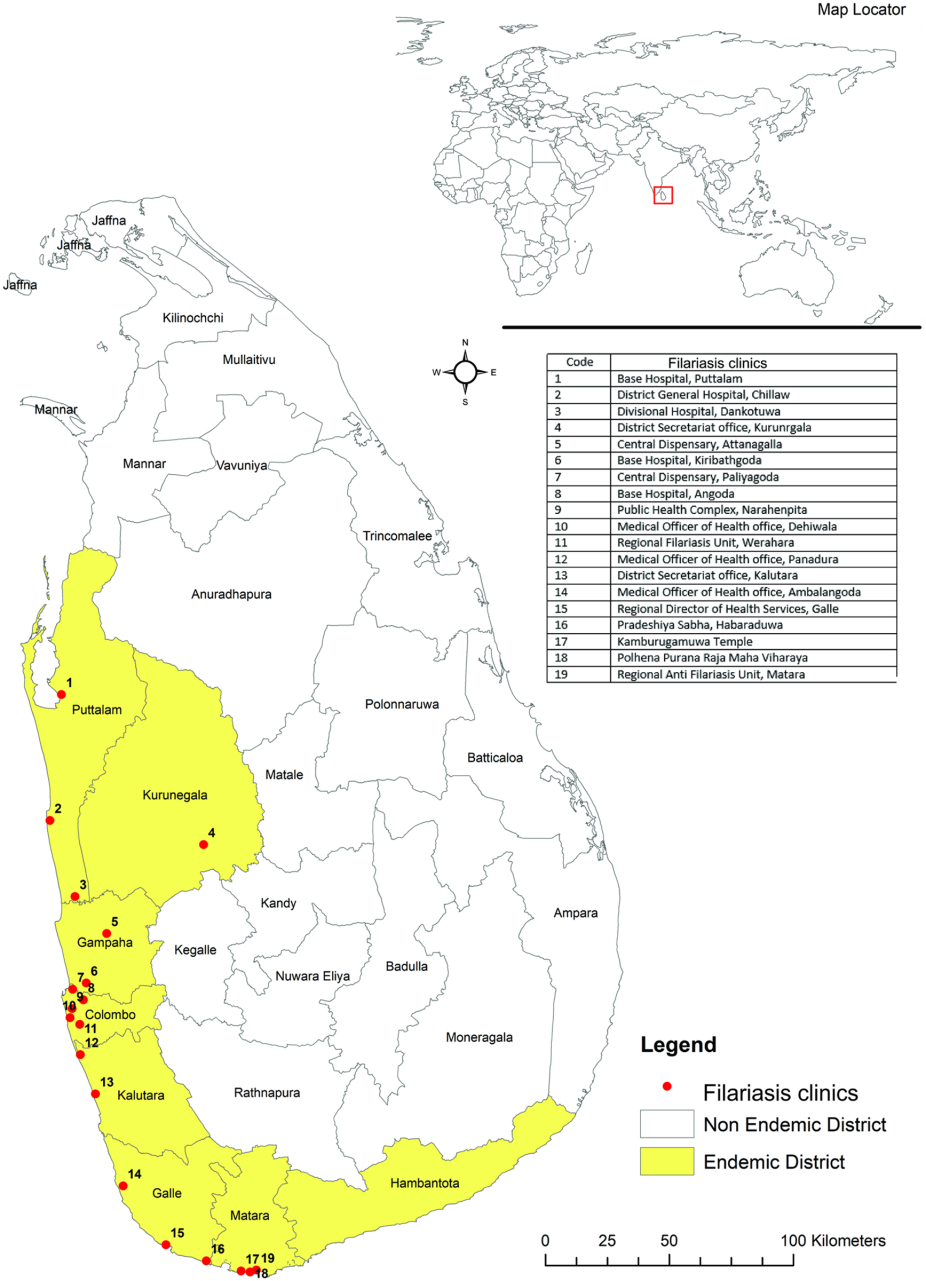
Distribution of filariasis clinics in the endemic districts of Sri Lanka.

A total of eight districts (Colombo, Gampaha, Kalutara, Galle, Matara, Hambantota, Puttalam and Kurunegala) belonging to three provinces (Western, Southern and North Western) in Sri Lanka were identified as endemic for LF during the elimination program (see Fig 1). Post-MDA surveillance in the endemic region revealed low-level persistence of bancroftian filariasis in a few areas and re-emergence of brugian filariasis after four decades [16], both of which require continued individual treatment.

Reduction of microfilaria rates below the target threshold of 1%, following five successful rounds of MDA (DEC + albendazole), enabled the submission of the elimination dossier in 2015. In 2016, Sri Lanka received validation from WHO of having eliminated LF as a public health problem [17]. Given the high profile efforts required to interrupt transmission, morbidity management has been given rather lower priority.

The current status of the MMDP program was assessed using the SWOT analysis tool, within the context of recent validation of LF elimination in Sri Lanka. SWOT analysis is defined as an examination of an organization’s internal strengths and weaknesses, the opportunities for growth and improvement, and the threats posed by the external environment to its survival [18]. This process was originally designed for use in the corporate sector and although novel, is gaining recognition for use in healthcare. Ideally SWOT analysis includes a comprehensive review of healthcare literature, in-depth data analysis and input from a panel of experts [18]. Findings from the analysis are sorted into four broad categories; strengths, weaknesses, opportunities and threats. This tool provides a framework for reviewing the program in an inter-disciplinary manner and brings the organization into balance with the external environment and maintains that balance over time. The goal of the SWOT analysis was to evaluate the MMDP program and services with the intent of providing recommendations for maximizing organizational performance with the limited resources available for achieving the goals of the MMDP program, i.e., to provide access to basic care for all affected by lymphatic filarial disease.

## Methods

In order to identify the strengths and weaknesses within the MMDP program and the opportunities and threats from outside the AFC and its MMDP program, a situation analysis was carried out in June 2017. The national Anti-Filariasis Campaign (AFC) is responsible for implementation of disease prevention and control strategies, which include conduct of filariasis clinics which are the main MMDP service providers. Data was collected for the SWOT analysis by reviewing LF data published in reports of the AFC, by in-depth interviews with identified key informants of the AFC and Regional Filariasis Control Units (RFCU), site-visits and focus group discussions with service providers of MMDP (filariasis clinics). Facts extracted from peer reviewed publications on patient based surveys and personal communications were also included in the analysis.

The key-informants included a Specialist Community Physician, Medical Officer, Public Health Nursing Sister, Public Health Inspector identified from the AFC (n = 4), and the RFCUs (Medical Officers, Public Health Field Officers and Public Health Inspectors, n = 6). Two open ended questions were included into interviews and group discussions to evaluate service performance and perceived challenges to MMDP program, namely, practices implemented for management of lymphoedema in their respective units and the challenges and obstacles faced by the respective units in implementing the program activities, especially those perceived to be linked to LF elimination status. Knowledge and adherence to disease management guidelines, adequacy of staff and vacant cadre positions, staff training and supervision and, the extent of political commitment for the MMDP program were also areas that were probed at interviews and group discussions. All interviews and discussions were conducted by persons external to the AFC and recorded with the consent of the informants to minimize data loss during transcription.

Information on clinic performance was extracted from the annual bulletins of the AFC, and lymphoedema management practices of clinic attendees and reasons for underutilization of clinics were extracted from peer reviewed publications, conference proceedings and personal communications. Existing external resources for improving the program were explored and identified as opportunities. The information thus gathered was evaluated and categorized into four broad themes: strengths, weaknesses, opportunities and threats.

## Results

### The organization of the national disease control program

The AFC was established in 1947 to reduce the burden of LF in Sri Lanka. It enabled LF elimination status by its early commitment to annual MDA, and has been documented as one of the finest LF elimination programs [19]. The AFC is headed by a director, supported by a deputy. It has cadre provision for many different categories of health personnel: specialist community physicians, medical officers, nursing sisters, public health inspectors, field assistants, laboratory staff, entomologists and assistants and administrative personnel (officers and support staff).

Activities related to filariasis control and morbidity management launched by the AFC were disseminated to the provinces via the RFCU. RFCUs have been established in seven of the eight endemic districts. The RFCUs, together with the AFC, continued with xenomonitoring guided enhanced surveillance to trace residual foci of infection and treated such persons with a 12-day regimen of DEC combined with a stat dose of albendazole and provided morbidity management services for lymphoedema patients. The AFC provided technical expertise, conducted staff training programs for new recruits and acted as the central body coordinating activities between the center and the periphery. The surveillance and morbidity data were submitted quarterly by RFCUs to the AFC, which compiled and disseminated the data in the form of quarterly and annual bulletins.

### Strengths of the MMDP program

#### Filariasis clinics

The network of well-established filariasis clinics (a total of 19) (Fig 1) that provided free at-the-point-of-use care to thousands of lymphoedema and elephantiasis patients in seven of the eight endemic districts was a major strength. All clinic sessions were held on weekdays (excluding public holidays), as half-day sessions, on a biweekly, weekly or monthly frequency, depending on the locality. A total of 711 and 697 clinic sessions were held in 2015 and 2016 respectively, at which 9,165 and 8,840 symptomatic cases respectively, were managed (Table 1) [20, 21]. These clinics were conducted by the staff of the RFCUs or AFC. Trained staff (medical officers and a public health nursing sister) experienced in LF morbidity alleviation conducted the morbidity management activities in these clinics.

**Table 1:** Lymphatic filariasis morbidity data in Sri Lanka as per Statistical Bulletins of the Anti-Filariasis Campaign, 2015–16.

Total number of clinic sessions		Year 2015	Year 2016
		711	697
Number of first visits	**Lymphoedema with acute attacks of cellulitis**	41	63
	**Lymphoedema without acute attacks of cellulitis**	816	690
	**Total number of new lymphoedema cases**	857	753
	Hydrocele/ TPE*	1/1*	5/4*
Number of subsequent visits	**Lymphoedema with acute attacks of cellulitis**	197	245
	**Lymphoedema without acute attacks of cellulitis**	8,111	7,842
	**Total number of past lymphoedema cases**	8,308	8,087
Total number of lymphoedoema cases		9,165	8,840

*TPE Tropical Pulmonary eosinophilia

The clinic activities included provision of care for acute inflammatory episodes, chronic manifestations and provision of chemoprophylaxis. Medications provided included oral antibiotics [penicillin, cloxacillin, erythromycin, co-amoxiclav (amoxacillin/clavulanic acid)], anti-filarials (DEC and albendazole), topical medications (antibiotics such as soframycin, antifungals such as miconazole and tioconazole, and steroids such as hydrocortisone), and antiseptics (potassium permanganate). Medications and bandages were provided free of charge and cases requiring more intensive management were referred for in-ward care. The most important service component was the provision of current, up to date, health education for clinic attendees and their care-givers on management of lymphoedema in the form of i) verbal advice (one-to-one talk) ii) demonstrations iii) provision of booklets or pamphlets in native language published jointly by the Ministry of Health and AFC under the sponsorship of the WHO entitled “New hope for people with lymphoedema” which depicted the recommended practices in picture form along with explanatory texts.

Published studies done among clinic attendees on lymphoedema management knowledge and practices, summarized below, provided evidence on the important role played by these clinics in morbidity management [22, 23]. Filariasis clinic attendees surveyed over a decade ago in the districts of Colombo and Gampaha revealed that the majority washed their limbs on a daily basis with soap and water (Colombo 75.5% and Gampaha 89.4%) while around half of the population practiced limb elevation (Colombo 46.6% and Gampaha 50%) and only a minority practiced recommended limb exercises (Colombo 14.7% and Gampaha 6%) [23, 24]. Usage of footwear varied in the two districts (Colombo 89.6% and Gampaha 48.5%) [23, 24]. Over half of the clinic attendees surveyed in Colombo (55.8%) made efforts to reduce trauma to the limbs and a few (10.4%) applied bandages [23].

#### Hospital surgical units

Patients who presented with hydroceles to primary health care personnel were referred to hospitals of patient preference, for hydrocelectomy on an in-patient basis. Referrals were made by general practitioners, consultants, hospital staff, RFCUs. Alternatively, the patients presented themselves on a first-contact basis for surgical intervention. State sector hospitals provided surgical care free of charge while private hospitals provided service on a fee levying basis. A total of 44 state sector hospitals within the LF endemic region of Sri Lanka were equipped with surgical units and theaters with facilities to perform hydrocelectomies [24]. The AFC had minimal influence on this aspect of morbidity management as hydroceles were directly referred to institutions equipped with surgical facilities. Data on hydrocelectomies performed in the preceding year in state medical institutions was collected by the AFC via requests sent to respective health institutions but was incomplete as some surgical facilities were non-compliant. Accordingly, at least 754 and 812 hydrocelectomies were performed in the years 2015 and 2016 in state hospitals of Sri Lanka (personal communication, AFC). Information on the probable cause of the hydroceles was unavailable. Severe ADLA episodes requiring administration of intravenous antibiotics referred from clinics or primary health care personnel were also managed on an in-patient basis in hospital facilities.

### Weaknesses in the MMDP program

Nine of the ten interviewees identified staff shortages as the main weakness. At the time of the analysis, there were vacant positions in all categories of staff (medical officers, field officers, entomological officers, laboratory technicians and labourers) with significant gaps in expertise (medical parasitologist, health educationists) within the work force of the AFC and RFCUs.

Lack of complete coverage of the entire endemic region was a major deficiency. One endemic district in the Southern Province (Hambantota district) lacked LF morbidity alleviation services. This was seen as an unacceptable situation, particularly because those affected with lymphoedema and elephantiasis have multiple issues associated with mobility (worsening of oedema, pain and discomfort, unwillingness to use public transport due to social stigma and shame) which would deter them from seeking treatment from distant clinics [5, 25]. In some districts that did have RFCUs and Filariasis Clinics, the accessibility of services was somewhat deficient, as the frequency of clinic sessions was low (weekly in Kurunegala) [26]. Limiting clinic sessions to weekdays also reduced their accessibility to patients who were employed. Morbidity management for hydroceles was provided by the state health sector and was thus accessible to all affected. However, little information on hydrocelectomies was available to the MMDP program, which was identified as a rectifiable weakness.

Although the advantages of community home-based care (CHBC) in LF morbidity alleviation have been well documented [23, 25], it is yet to be implemented in Sri Lanka. Lack of indicators to measure successful management of morbidity (e,g., stage regression of lymphoedema, reduction of frequency of ADLA, improvement in quality of life) and similar targets for evaluating program success, was another deficiency identified within the program [27]. Guidelines or care-pathways for disability management and rehabilitation of those with disability were also yet to be established.

### Threats to the MMDP program

Some of the challenges were consequent to achievement of LF elimination status. The most imminent threat perceived by eight of ten key informants (AFC), was the diversion of staff and resources of the AFC and RFCUs for control of other vector-borne infections such as dengue, which were regarded as more important due to its associated mortality. Loss of alliances and funding was another factor that was regarded by majority of interviewees (70%) as hindering expansion and strengthening of the MMDP program, especially its rehabilitation component.

The available treatment facilities were greatly underutilized, partly attributed to lack of awareness of their existence even among the medical community. Other reasons for underutilization of clinic services were, lack of confidence with regard to efficacy of recommended therapy (experienced by 83% of a case cohort of lymphoedema patients in Matara [28]), the costs associated with attending clinics (reported by Perera *et al* and Yahatugoda *et al* [5, 25] and the social stigma of being labelled as a case of filariasis by attending clinics (reported by the majority of females (71%) and 42% of males in the Matara study [28] as well as in others[5].

Another challenge to scaling up the morbidity alleviation programme was the dearth of evidence on how best to integrate the services into the existing health systems.

### Opportunities for strengthening the MMDP program

Strong high level commitment within the Ministry of Health for LF morbidity alleviation and disability management was regarded as essential for sustaining and strengthening the MMDP program. Integrating filariasis management with management of other chronic diseases such as diabetes, leprosy or non-filarial lymphoedema (establishment of lymphoedema management centers rather than filariasis clinics) was recommended as it would be cost-effective. Such a strategy would maximize the use of limited resources as well as overcome the social stigma of being labelled as a case of ‘filariasis’, by reason of attending filariasis clinics.

Publicity campaigns to raise awareness of treatment centers would be a simple way to improve their utilization. This could be achieved by displaying posters and banners at community centers, hospitals and other health care facilities. Establishment of referral systems through primary health care providers (medical officers, general practitioners, and field staff) would further improve utilization of clinics.

Primary health care providers such as general practitioners, medical officers at hospital out-patients-departments and even consultants, require to be up-dated on current lymphoedema management strategies to ensure provision of appropriate care.

Collaborations with Non-Governmental Organizations and research groups would provide opportunities for national program managers to obtain much needed funds and expertise for the program. Inter-ministerial collaborations for provision of rehabilitation facilities that were beyond the purview of the Ministry of Health (eg, Department of Social Services under Ministry of Social Empowerment Welfare and Kandyan Heritage) was identified as another opportunity to rehabilitate those with disability. The importance of conducting evidence based and operational research for optimizing management (e.g. newly adopted triple therapy in the management of lymphoedema) and service delivery (integration of MMDP into primary health care system) is emphasized.

## Discussion

The MMDP program requires a broad strategy with primary, secondary and tertiary prevention [13]. Primary prevention of morbidity is by MDA while secondary prevention is aimed at preventing progression of lymphoedema to elephantiasis by minimizing or preventing ADLA episodes and lymph stasis. The filariasis clinics focus on secondary prevention whereby patients are made aware of the importance of adhering to simple hygiene based measures such as regular washing with soap and water, drying, management of entry lesions with topical medications, wearing appropriate footwear and prevention of lymph stasis by elevation, compression bandages and recommended exercises.

Although primary and secondary prevention of LF morbidity is functional in most of the endemic region of Sri Lanka, shortcomings exist. Similar deficiencies, particularly those associated with service delivery, have been reported in neighboring Pondicherry [15].

Establishment of a RFCU and a filariasis clinic in the district of Hambanthota is strongly recommended. In addition, service delivery may be improved by increasing the frequency of clinics (district of Kurunegala) and establishing week-end clinics to cater to the needs of the employed.

Tertiary prevention includes psychological and socioeconomic support for people with disability, and ensuring that those affected have equal access to rehabilitation, opportunities for health education and income generation [13]. Such interventions require clearly committed resources and funds which is why they are yet to be implemented in the National MMDP programme.

The potential limitations of this analysis, which was based on data derived from multiple sources, is acknowledged and more patient oriented research on the subject is recommended with inclusion of higher participant numbers.

The aim of the GPELF is to eliminate LF as a public health problem. The health impact of LF is the morbidity caused by this infection. However, programmatic targets have focused on interrupting transmission rather than on alleviation of morbidity. LF control program targets should include morbidity indicators which go beyond measuring access to care, to indicators that link success to the extent of morbidity alleviation [27]. As we approach 2020, and WHO and the GPELF take stock of progress towards its goals, review of the indicators used to measure success, would be very timely.

### Conclusions

The recommended minimum package for morbidity alleviation of LF is functional in most parts of the endemic area of Sri Lanka. However, the MMDP program has significant weaknesses and threats that need to be addressed, in order to enable national program managers to scale up and strengthen the program.

## References

[1] WHO. Bridging the Gap, The World Health Report, World Health Organization. 1995; Geneva, Switzerland.

[2] Ramaiah KD, Vijay Kumar KN, Ramu K, Pani SP, Das PK. Functional impairment caused by lymphatic filariasis in rural area of south India. Trop Med Int Health. 1997; 2: 832–838 9315041 10.1046/j.1365-3156.1997.d01-406.x

[3] Krishna Kumari A, Harichandrakumar KT, Das LK, Krishnamoorthy K. Physical and psychosocial burden due to lymphatic filariasis as perceived by patients and medical experts. Trop Med Int Health. 2005; 10: 567–573. doi: 10.1111/j.1365-3156.2005.01426.x 15941420 10.1111/j.1365-3156.2005.01426.x

[4] Wijesinghe RS, Wickremasinghe AR. Physical, psychological, and social aspects of quality of life in filarial lymphedema patients in Colombo, Sri Lanka. Asia Pac J Public Health. 2015; (2): NP 2690–701. doi: 10.1177/1010539511434140 Wijesinghe RS 10.1177/1010539511434140 22308536

[5] Perera M, Whitehead M, Molyneux D, Weerasooriya M, Gunatilleke G. Neglected patients with a neglected disease? A qualitative study of lymphatic filariasis. PLoS Negl Trop Dis. 2007; (2):e128 doi: 10.1371/journal.pntd.0000128 18060080 10.1371/journal.pntd.0000128 PMC2100378

[6] Gyapong JO, Gyapong M, Evans DB, Aikins MK, Adjei S. The economic burden of lymphatic filariasis in northern Ghana. Ann Trop Med Parasitol. 1996; 90: 39–48. 8729626 10.1080/00034983.1996.11813024

[7] Ramaiah KD, Ramu K, Guyatt H, Vijay Kumar KN, Pani SP. Direct and indirect costs of the acute form of lymphatic filariasis to households in rural areas of Tamil Nadu, south India. Trop Med Int Health. 1998; 3: 108–115 9537272 10.1046/j.1365-3156.1998.00208.x

[8] Ramaiah KD, Guyatt H, Ramu K, Vanamail P, Pani SP, Das PK. Treatment costs and loss of work time to individuals with chronic lymphatic filariasis in rural communities in South India. Trop Med Int Health. 1999; 4: 19–25 10203169 10.1046/j.1365-3156.1999.00351.x

[9] Babu BV, Nayak AN. Treatment costs and work time loss due to episodic adenolymphangitis in lymphatic filariasis patients in rural communities of Orissa, India. Trop Med Int Health. 2003; 8: 1102–1109. 14641845 10.1046/j.1360-2276.2003.01146.x

[10] Mukhopadhyay AK. Lymphatic filariasis in Andhra Pradesh Paper Mill Colony, Rajahmundry, India after nine rounds of MDA Programme. J Vector Borne Dis. 2010; 47: 55–57. 20231775

[11] Fenwick A. The global burden of neglected tropical diseases. Public Health. 2012;126 (3):233–236. doi: 10.1016/j.puhe.2011.11.015 22325616 10.1016/j.puhe.2011.11.015

[12] WHO position statement: Managing morbidity and preventing disability in the Global Programme to Eliminate Lymphatic Filariasis. World Health Organization Geneva Switzerland 2011. WHO/HTM/NTD/PCT/2011.8 22191103

[13] WHO. Lymphatic filariasis: managing morbidity and preventing disability; an aid- memoir for national programme managers Global Programme to Eliminate Lymphatic Filariasis. World Health Organization 2013; Geneva, Switzerland

[14] WHO. Global programme to eliminate lymphatic filariasis: progress report, 2016 WEEKLY EPIDEMIOLOGICAL RECORD, NO. 40, October 2017, 92, 589–608. http://www.who.int/wer

[15] Kumari AK, Yuvaraj J, Das LK. Issues in delivering morbidity management for lymphatic filariasis elimination: a study in Pondicherry, South India. Sci World J. 2012; 10.1100/2012/372618 PMC3361224 22654597

[16] Mallawarachchi CH, Nilmini Chandrasena TGA, Premaratna R, Mallawarachchi SMNSM, de Silva NR. Human infection with subperiodic Brugia spp. in Gampaha district, Sri Lanka: a threat to filariasis elimination status? Parasit Vectors 2018;11(1):68 doi: 10.1186/s13071-018-2649-3 29378620 10.1186/s13071-018-2649-3 PMC5789669

[17] WHO Country Office for Sri Lanka. WHO officially declares Sri Lanka filariasis free. Colombo: World Health Organization; 2016 http://www.searo.who.int/srilanka/documents/WHO_officially_declares_Sri_Lanka_filariasis_free/en/ [accessed 10 October 2017].

[18] Harrison JP. Strategic Planning and SWOT Analysis In Essentials of Strategic Planning in Healthcare. Health Administration Press 2010 pp 91–97. http://www.ache.org/pdf/secure/gifts/Harrison_Chapter5.pdf

[19] Rao RU, Nagodavithana KC, Samarasekara SD, Wijegunawardana AD, Premakumara WD, Perera SN, et al A comprehensive assessment of lymphatic filariasis in Sri Lanka six years after cessation on mass drug administration. PLoS Negl Trop Dis. 2014; 8(11):e3281/ doi: 10.1371/journal.pntd.0003281 25393404 10.1371/journal.pntd.0003281 PMC4230885

[20] AFC. Annual Statistical Bulletin 2015. Anti Filariasis Campaign, Ministry of Health

[21] AFC. Annual Statistical Bulletin 2016. Anti Filariasis Campaign, Ministry of Health Sri Lanka.

[22] Chandrasena TN, Premaratna R, de Silva NR. Lymphoedema management knowledge and practices among patients attending filariasis morbidity control clinics in Gampaha District. Sri Lanka. Filaria J. 2004; 3(1):6 doi: 10.1186/1475-2883-3-6 15287989 10.1186/1475-2883-3-6 PMC514715

[23] Wijesinghe RS, Wickremesinghe AR, Ekanayake S, Perera MSA. Efficacy of a limb-care regime in preventing acute adenolymphangitis in patients with lymphedema caused by bancroftian filariasis in Colombo, Sri Lanka. Ann Trop Med Parasitol, 2007;101(06):487–497 17716431 10.1179/136485907X193806

[24] Medical Statistics Unit. Annual Health Bulletin (2015) Sri Lanka. Ministry Of Health Nutrition and Indigenous Medicine. Sri Lanka. www.health.gov.lk/moh_final/english/public/elfinder/files..../AHB%202015pdf (Accessed 2nd November 2017)

[25] Yahatugoda TC, Weerasooriya MV, Samrawickrema WA. Elimination of lymphatic filariasis in Sri Lanka: How an independent research group supported the programme www.filariasis.org/documents/SriLankaStory. [accessed 10th October 2017]

[26] Anti Filariasis Campaign. www.filariasiscampaign.health.gov.lk. [accessed 10th October 2017]

[27] Deribe K. Neglected tropical disease targets must include morbidity. Lancet Glob Health. 2015;3(10):e596 doi: 10.1016/S2214-109X(15)00185-0 26385299 10.1016/S2214-109X(15)00185-0 PMC4594778

[28] Yahatugoda TC, Wickremasinghe D, Weerasooriya MV, Samarawickrema WA, Lymphoedema and its management in cases of lymphatic filariasis: the current situation in three suburbs of Matara, Sri Lanka, before the introduction of a morbidity-control programme. Ann Trop Med Parasitol, 2005; 99(5):501–10 doi: 10.1179/136485905X46450 16004709 10.1179/136485905X46450

